# Editorials

**Published:** 1912-03

**Authors:** 


### EDITORIAL

The Business Office of the *Journal-Record* is 1 1-2 to 5 1-2 South Broad Street.
The Editorial Office is 1014-15 Century Building.
Address all Business Communications to *Journal-Record of Medicine*, 1 1-2 to 5 1-? South Broad Street.
Make remittances payable *to The Journal-Record of Medicine.*

On matters pertaining to the Editorial and Original Communications, address Edgar G. Ballenger, M. D., Atlanta, Ga.
Reprints of Original Articles will be furnished at cost price. Requests for the same should always be made in THE MANUSCRIPT.

We will present, postpaid, on request, to each contributor of an original article, twenty (20) marked copies of *The Journal-Record of Medicine* containing such article.

### THE FIRST AMERICAN BOOK ON PELLAGRA.

While innumerable short articles on pellagra have flooded the medical press of late, and while a translation of Dr. Marie's work has appeared in this country, the first volume on pellagra from an English or American writer has just now come from the press of W. B. Saunders Co., Philadelphia, under the title of "Pellagra—An American Problem," by George M. Niles, M. D., Professor of Gastro-Enterology and Therapeutics in the Atlanta School of Medicine.

The publication of an American book on pellagra is most timely; the increasing prevalence and virulence of the disase, together with its more or less distinct variations in this country from the types studied abroad, make the demand imperative for an authoritive, comprehensive and up-to-date work on pellagra. This volume from the facile pen of Dr. Niles seems to meet the demand with not only ability, but with cleverness and grace as well.

The work comprises ten chapters entitled as follows:

1. General Consideration, Historic and Otherwise.
2. Pellagra in the United States.
3. A Discussion of the Etiology of Pellagra.
4. Symptomatology and Clinical Course of Pellagra.
5. Clinical Reports and Description of Cases of Pellagra from Different Sources.

6. Pathology and Morbid Anatomy of Pellagra.

7. Diagnosis, Course and Progress, and Prognosis of Pellagra.

8. The Treatment of Pellagra—A Discussion of Different Methods.

9. The Prophylaxis of Pellagra.

10. Description of Some Recent Experiments on Animals, and Deductions Therefrom.

Dr. Niles frankly acknowledges that we have not solved the problem of the causation of pellagra; yet, he gives fair and courteous consideration to all theories thus far advanced and expresses his own leaning towards the bad-corn-hypothesis. Whether right or wrong in this preference, he is certainly in company with the majority of students of the disease.

It is gratifying to note the full consideration allotted in this book to the nervous phenomena of pellagra—a consideration covering nearly thirty pages. However predominant the skin lesions may appear to the superficial observer, the reviewer is convinced that the greatest damage sustained by the victim of pellagra is in the nervous tissues, and that probably both dermal and gastro-intestinal lesions are trophic in nature and, therefore, secondary to the disturbances of the nervous system.

From the standpoint of medical literature in general, this work by Dr. Niles is distinctive in its literary charm; and seldom in these days of technical jargon and scientific circumlocution do we run across so refreshing an example of linguistic grace and lucidity.

But more important still is the optimism exhibited by the author in his chapters on prophylaxis and treatment. Dr. Niles is not a victim of pellagraphobia. On the other hand, he takes a hopeful view of those cases which may be taken in hand early and given the benefit of the hygienic, dietetic and medicinal measures.

The illustrations are many and excellent, and Messrs. Saunders have sustained their reputation for high-grade mechanical work by rendering Dr. Niles' work into a most attractive volume.

HANSELL CRENSHAW, M. D.

### MORTALITY STATISTICS OF 1910.

The mortality statistics of 1910, issued by the Department of Commerce and Labor, Bureau of the Census, under the direction of E. Dana Durand, presents some advance information which should be of great value to sanitarians. We regret that none of the Southern States has compiled sufficiently correct statistics to be included in this report, except North Carolina, which gave statistics from municipalities having 1,000 or more inhabitants. Especial interest is shown in the North Carolina reports, which are in compliance with a new law requiring reports from municipalities having a population of 1,000 or over. It is sincerely hoped that this is the beginning of effective registration in the South.

The administration and enforcement of the law is made the duty of the State Board of Health. The practical difficulties in the way of attaining effective State registration in some parts of the South are so great that any plan likely to secure registration, according to standard and uniform methods, of a considerable proportion of deaths deserves careful consideration.

The total number of deaths reported during 1910 from the registration area was 805,412; of these 154, 373 were infants under one year of age. Tuberculosis still leads as the most important cause of death, being responsible for 10.7 per cent. of the total. The other chief causes come in the following order: Organic diseases of the heart; diarrhoea and enteritis; pneumonia; acute nephritis; accident and malignant growths.

As regards age-periods, diarrhoea and enteritis caused the greatest number of deaths under 2 years; diphtheria and croup between 3 and 4; tuberculosis between 10 and 50, while organic heart disease is the most important cause after 50 years of age.

Suicide starts with 1.1 per cent. of all deaths at the 10 to 19 years age-period, increasing to 2.8 at 20 to 29 years, then decreasing to 2.6 at 30 to 39 years, 2.3 at 40 to 49 years, 1.8 at 50 to 59 years, stopping with 1.0 per cent. of all deaths at the age-period of 60 to 69 years.

Homicide begins with 1.7 per cent. of all deaths at the ageperiod of 20 to 29 years, 1.2 at 30 to 39 years, and 0.6 at 40 to 49 years.

Pneumonia (lobar and undefined) claims 5.5 per cent. of all deaths of infants under 1 year of age, 10.1 at 1 year, 10.0 at 2 years, 8.1 at 3 years, 7.5 at 4 years, 6.6 of all deaths in the age-period of under 5 years, 6.3 at 5 to 9 years, 6.6 of all under 10 years, 5.7 at 10 to 19 years, 5.6 at 20 to 29 years, 7.1 at 30 to 39 years, 7.8 at 40 to 49 years, 7.6 at 50 to 59 years, 7.1 at 60 to 69 years, 6.5 at 70 to 79 years, 5.7 at 80 to 89 years, and 5.1 at 90 years and over.

Appendicitis begins with 1.0 per cent. of all deaths of children at 4 years of age, 3.2 of all at 5 to 9 years, 4.7 at 10 to 19 years, 2.0 at 20 to 29 years, 1.4 at 30 to 39 years, and ends with 1.0 at 40 to 49 years.

Acute Nephritis, Bright's disease, caused from 3.4 to 6.3 per cent. of the deaths at the 10 year age-periods from 10 to 39 years of age, but it increased to 9.5 per cent. at 40 to 49 years, advancing again to 11.7 per cent. at 50 to 59 years, still increasing to 12.1 per cent. at 60 to 69 years, then falling to 11.1 per cent. at 70 to 79 years, decreasing again to 8.5 per cent. at 80 to 89 years, and finally becoming 5.4 per cent. at 90 years and over.

Cancer is not charged with any percentages in the age periods up to 19 years of age, but it formed 1.1 per cent. at 20 to 29 years, 3.8 per cent. at 30 to 39 years, 9.0 per cent. at 40 to 49 years, 12.1 per cent. at 50 to 59 years, 11.1 per cent. at 60 to 69 years, 7.8 per cent. at 70 to 79 years 4,5 per cent. at 80 to 89 years, and 2.2 per cent. at 90 years and over.

Typhoid fever caused 8.0 per cent. of the deaths at the age-period 10 to 19 years, but decreased to 6.0 per cent. at 20 to 29 years, to 3.2 per cent. at 30 to 39 years, to 1.8 per cent. at 40 to 49 years, to 1.1 per cent. at 50 to 53 years, there being none for the remaining age-periods.

Cerebral hemorrhage, or apoplexy, began its course with 0.7 per ent. of all the deaths at the 20 to 29 years age-period, then constantly increasing to 1.7 at 30 to 39 years, 4.0 at 40 to 49 years, 7.5 at 50 to 59 years, 10.7 at 60 to 69 years.

More deaths occurred in March than in any other month.

While tuberculosis is shown to be increasing very slightly, cancer increased from 738 per 100,000 in 1909 to 762 in 1910. Other countries show also a similar increase. The increase is probably due in part to increased care in diagnosis, in part to a larger proportion of persons who reach an advanced age and also to an actual increase in the prevalence of cancer.

---

### MOUSE TUMORS AND SELENIUM.

That chemo-therapy, as elaborated by Paul Ehrlich, would continue to develop in other fieldst than in diseases caused by spirochetes has been doubted only by the worst pessimists. The scientific principles underlying the discovery of salvarsan should show the way to find other similar remedies. Following this trail Dr. Wassermann and his co-workers appear to have made a remarkable observation that "mouse tumors" were favorably influenced by a combination of selenium and eosin. This remedy has a selective action for the cancerous mass" and caused its liquifaction after three injections. Of course, the discovery has not yet reached the stage when it is available as a remedy for cancer, but is now being studied with the view of finding a preparation free from toxic effect yet possessing the virtues of selenium. Many of the mice treated died, but some lived and appeared to be cured. With such able men working to find a suitable chemical combination—free from deleterious effects—we may at least wish them success. Nothing yet has been done which can be utilized in the treatment of cancer. Unfortunately, the lay press has given much space to sensational and unwarranted reports. Soon quacks and vampires will be preying upon the gullible public with fake remedies purporting to be Wassermann's discovery.

---

### THE GEORGIAN HOSPITAL.

The substantial strides Atlanta is making as a medical and surgical center is clearly demonstrated by the improvement in her hospitals and the new ones being built. One recently opened and beautifully fitted throughout with every modern convenience for the treatment of disease is the Georgian Hospital, which can accommodate about 45 patients.

The hospital was founded by Mr. D. M. Matthews and is located at 235 Capitol Avenue, corner of Richardson Street. It is non-sectarian; any city doctor can send his patients there and at the same time continue the treatment himself. The hospital is four stories in height, and throughout the entire building no expense has been spared to make it as near ideal in arrangement as possible. The lower floor is taken up with office, parlors, dining rooms and lounging rooms for the physicians. On the second and third floors are the rooms for patients, beautifully fitted up, some with private baths. The operating rooms, of which there are two, are located on the top floor and are as fine as any in the South, very large windows on all sides giving an abundance of light; adjoining them is the wash room, sterilizing room, anaesthetic room and bath room.

The officers of the hospital staff are Dr. J. H. Bradfield, president; Dr. W. L. Gilbert, vice president; Dr. R. R. Daly, secretary; Dr. J. S. Todd, general consultant.

